# Building the Field of Health Policy and Systems Research: Social Science Matters

**DOI:** 10.1371/journal.pmed.1001079

**Published:** 2011-08-23

**Authors:** Lucy Gilson, Kara Hanson, Kabir Sheikh, Irene Akua Agyepong, Freddie Ssengooba, Sara Bennett

**Affiliations:** 1 School of Public Health and Family Medicine, University of Cape Town, Cape Town, South Africa; 2 Department of Global Health and Development, London School of Hygiene and Tropical Medicine, London, United Kingdom; 3 Public Health Foundation of India, New Delhi, India; 4 Ghana Health Service/School of Public Health, University of Ghana, Accra, Ghana; 5 School of Public Health, Makerere University, Kampala, Uganda; 6 Health Systems Programme, Johns Hopkins Bloomberg School of Public Health, Baltimore, Maryland, United States of America

## Abstract

In the second in a series of articles addressing the current challenges and opportunities for the development of Health Policy and Systems Research (HPSR), Lucy Gilson and colleagues argue the importance of insights from the social sciences.

***PLoS Medicine* Series on HPSR**

Following the *First Global Symposium on Health Systems Research* in Montreux in November 2010, *PLoS Medicine* commissioned three articles on the state-of-the-art in Health Policy and Systems Research (HPSR). Three Policy Forum articles, authored by a diverse group of global health academics, critically examine the current challenges to the field and lay out what is needed to build capacity in HPSR and support local policy development and health systems strengthening, especially in low- and middle-income countries.

*Paper 1*. Kabir Sheikh and colleagues. Building the Field of Health Policy and Systems Research: Framing the Questions.

*Paper 2*. Lucy Gilson and colleagues. Building the Field of Health Policy and Systems Research: Social Science Matters.

*Paper 3*. Sara Bennett and colleagues. Building the Field of Health Policy and Systems Research: An Agenda for Action.

**Summary Points**

- All researchers hold a knowledge paradigm that frames their understanding of reality and of the functions and nature of research. Some disciplines are dominated by a particular paradigm and some are spread across paradigms.
- The criticisms that Health Policy and Systems Research (HPSR) is too context specific, does not offer clear lessons for policy makers, and is not rigorous are partly a reflection of differences in knowledge paradigms between those with predominantly clinical, biomedical, and epidemiological backgrounds, underpinned by a positivist paradigm, and those with social science backgrounds underpinned by a relativist paradigm.
- Health policies and systems are complex social and political phenomena, constructed by human action rather than naturally occurring. Relativist social science perspectives are, therefore, of particular relevance to HPSR as they recognise that all phenomena are in essence constructed through human behaviour and interpretation.
- Social science insights that can advance the science of HPSR include approaches to generalising from rich understanding of context; supporting policy learning; and enhancing research rigour and quality.

## Introduction

The first paper in this series on building the field of Health Policy and Systems Research (HPSR) in low- and middle-income countries (LMICs) [1] outlined the scope and questions of the field and highlighted the key challenges and opportunities it is currently facing. This paper examines more closely one key challenge, the risk of disciplinary capture—the imposition of a particular knowledge frame on the field, privileging some questions and methodologies above others. In HPSR the risk of disciplinary capture can be seen in the current methodological critique of the field, with consequences for its status and development (especially when expressed by research leaders).

The main criticisms are reported to be: that the context specificity of the research makes generalisation from its findings difficult; lack of sufficiently clear conclusions for policy makers; and questionable quality and rigour [2]. Some critique is certainly warranted and has come from HPS researchers themselves. However, this critique also reflects a clash of knowledge paradigms, between some of those with clinical, biomedical, and epidemiological backgrounds and those with social science backgrounds. Yet, as HPSR is defined by the topics and questions it considers rather than a particular disciplinary approach, it requires engagement across disciplines; indeed, understanding the complexity of health policy and systems demands multi- and inter-disciplinary inquiry [3].

To develop the science of HPSR it is, therefore, important to start by recognising the diversity of disciplinary perspectives, as well as shared concerns. Richer methodologies for addressing these concerns must then be developed. And, as health policies and systems are themselves social and political constructions, it is important to acknowledge the particular value of social science perspectives in the field. Each of these issues is addressed in the following sections, and they are considered further in paper three of the series [4].

## Knowledge Paradigms

**Figure 1:**
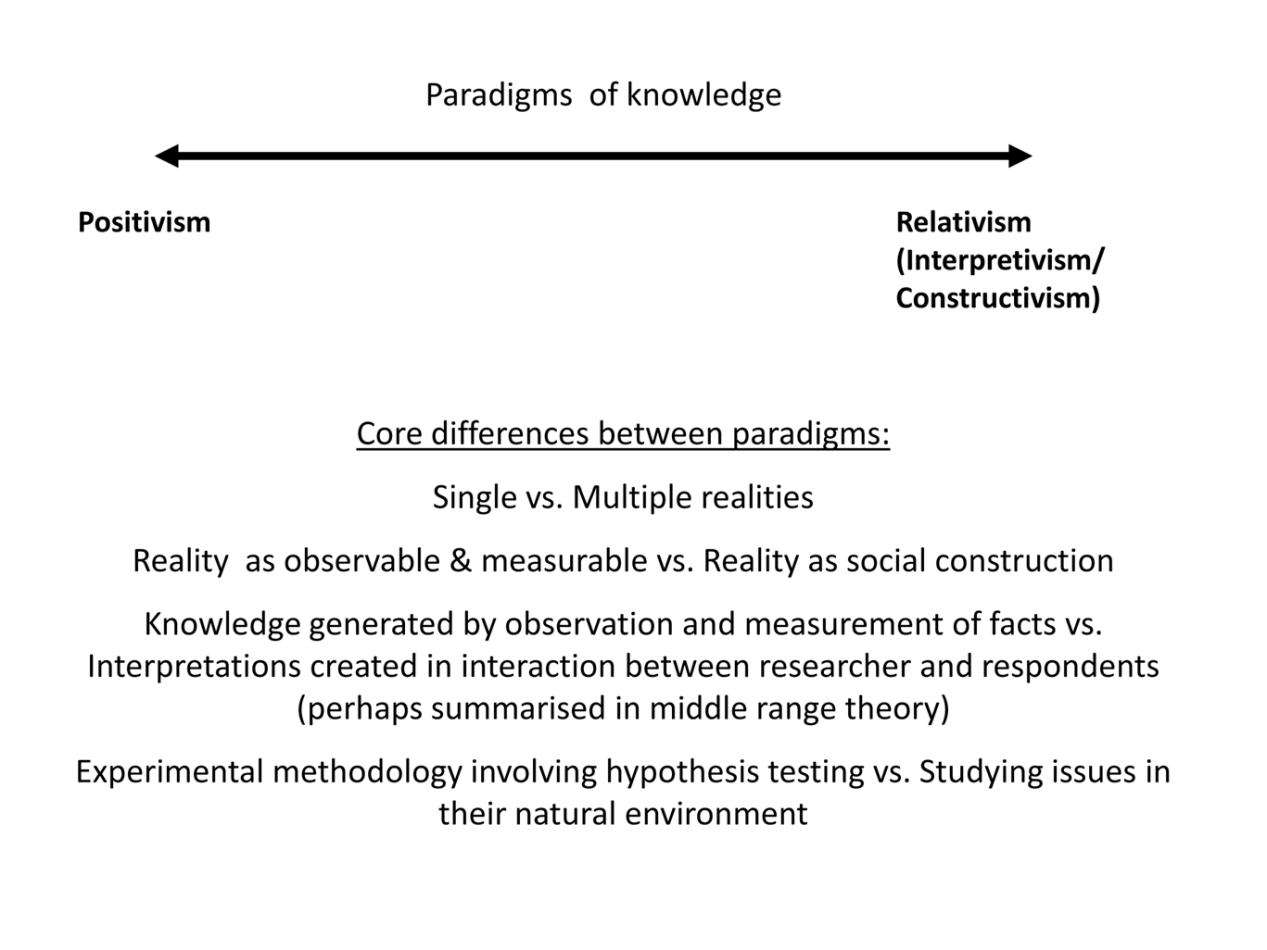
Core differences between knowledge paradigms.

Figure 1 characterises key areas of difference between the dominant knowledge paradigms that underpin the disciplines applied within HPSR. The figure deliberately polarises the paradigms to spark debate. Some disciplines are dominated by a particular paradigm and some are spread across paradigms.

The positivist worldview is reflected in much clinical, biomedical, and epidemiological, and some social science, research. This view starts from the same position as the natural and physical sciences. The phenomena being investigated comprise **a set of facts, a single reality** that can be **observed and measured** by the researcher without disturbing them. The central aim of research is to detect causal mechanisms through the deductive process of **testing hypotheses** derived from theory and past experience against empirical facts. At their simplest, such mechanisms represent the prediction that “x will cause y” in any other setting. Simple HPSR hypotheses might include, for example, “limited financial incentives cause low motivation” or “a lack of health facilities undermines access to health services.” Sometimes such hypotheses are tested through statistical analysis of secondary data [5]; sometimes studies are designed to allow hypotheses to be tested [6]. Indeed, the positivist perspective underpins the recent rise of **experimental methodology** in impact evaluation. As the emphasis in such studies is on measuring the magnitude of an intervention's impact, and ensuring that this estimate is unbiased, careful attention is paid to selecting an appropriate control group (randomized or otherwise) and controlling the influence of possible confounding factors. Much less emphasis is placed on understanding how the intervention works and which contextual or other factors mediate its impact.

Much social science work that is qualitative is located at the relativist end of the spectrum. Such research is essentially based on the understanding that the world around us is subject to human interpretation. Health policies and systems are, therefore, understood to be **constructed and brought alive** by social actors through the meaning they attach to (their interpretations of) their experiences. Whereas positivist researchers focus on facts and regularities (that is, causes and effects), relativist researchers see **interpretations** as the primary subject of inquiry, proposing that different interpretations of the same experience represent **multiple realities**. In this tradition, researchers study human behaviour in **everyday or natural settings**, generating qualitative data that are primarily analysed inductively to generate categories and explanations of experience. Such analysis also involves **interpretation by the researcher**, in interaction with respondents. It may be guided by, and/or generate, what is called **middle range theory**, i.e., ideas about how the world works, comprising categories and concepts derived from analysis, and suggestions about how they are linked together. Middle range theory may be tested against evidence through the process of analysis or highlights questions and ideas to be considered in future studies.

Relativist HPSR studies focus, for example, on how health system actors understand and experience particular services or policies [7], and what social and political processes, including power relations, influence them [8],[9]. The development and testing of middle range theory is also supported by studies that adopt a critical realist position. This knowledge paradigm falls somewhere in the spectrum between positivism and relativism, and is of growing interest in HPSR [10] (see FEMhealth, http://www.abdn.ac.uk/femhealth/). However, these sorts of questions are still only quite rarely addressed in the wider HPSR literature [1].

## Shared Concerns and the Value of Multiple Perspectives

Although HPS researchers from different disciplinary traditions have some difficulty understanding each other's perspectives, they also have some shared starting points: a common focus, health policies and systems, and a concern about how to strengthen health systems to benefit those being served by them. The complexity of the phenomena being investigated may also generate a willingness to think creatively about how to investigate issues. Therefore, HPS researchers tend not to fall at the extreme ends of the spectrum outlined in Figure 1—and this makes multi- and inter-disciplinary work more possible.

Review of existing HPSR work demonstrates, moreover, that bringing together research from different traditions generates broader and deeper understanding on the issues of focus. Box 1, for example, shows the breadth of questions that have been addressed around one critical HPS issue for LMICs, user fees; and the different papers examining the household level impacts of out of pocket payments together provide deeper and richer insights on these experiences than would come from one perspective alone.

**Box 1. Drawing on Different Perspectives to Understand and Explain Experiences of User Fee Policy Change in Low- and Middle-Income Countries**

Assessing household level impacts

*Positivist perspectives:*

- What is the impact of out of pocket payments on household poverty levels across countries?
  Cross-national statistical analysis [5] (health economics)
- What is the impact of user fee removal on aggregate patient utilisation and across different patient socioeconomic groups within one country?
  Before and after statistical analysis [24] (health economics)

*Relativist perspectives:*

- How do pocket payments combine with other influences over health-seeking behaviour to impact on the dynamics of household poverty?
  Mixed method study involving longitudinal household case studies [22] (development sociology, health economics)

Explaining policy implementation experiences

*Critical realist and relativist perspectives:*

- What political forces led to user fee introduction/removal, and why was equity neglected as a policy goal?
  Qualitative study [25] (social anthropology, policy analysis)
- How does the process of implementing user fee removal influence health worker morale?
  Multiple method study within overarching qualitative approach [26] (sociology, policy analysis)
- How is the process of implementing user fees, in interaction with other policies, influenced by wider societal forces?
  Ethnographic study [27] (anthropology)

## Learning from Relativist Social Science Perspectives

Health policies and systems are fundamentally shaped by political decision-making, whilst the routines of health systems are brought alive through the relationships among the actors involved in managing, delivering, and accessing health care, and engaged in wider action to promote health, including researchers [11]. In essence, therefore, health policies and systems are constructed through human behaviour and interpretation, rather than existing independently of them. As relativist social science perspectives see all phenomena as at least partially constructed in this way, they have particular value in building the methodological foundations of HPSR. Three contributions are discussed here: generalising from rich contextual understanding; supporting policy learning; and approaches to ensuring research rigour.

### Taking Account of Context in Drawing out Generalisations

Multiple contextual factors influence the working of health systems. Health worker motivation, for example, reflects a range of personal, organisational, and societal factors, including relationships with others, and itself influences many aspects of the provision of health care. Similarly, patients' decisions to use services, or adhere to treatment advice, are responses to many contextual factors: their own understandings of illness, and how best to treat it; advice received from friends and family; past experience of health providers; the availability of cash to cover costs; and the gender dynamics influencing household decision-making. There are also multiple interpretations of the same experience as different people bring different contexts to bear on its interpretation. Health workers, for example, respond differently to the same financial incentive, and patients vary in their response to treatment advice. The causal mechanisms underpinning the changes brought about by new health policies or health system interventions are, thus, complex.

As a result, investigation of HPS issues demands research that seeks to understand and explain experiences by reference to the many layers of their context, whilst acknowledging the often quite different interpretations of experience across people. Reducing relevant contextual factors to a set of simple quantifiable measures for statistical analysis is, simply, difficult. On the other hand, case study research, widely used in organisational and political science work, supports the “thick descriptions” of particular experiences situated within their context that allow understanding and explanations of the phenomena of focus by reference to that context [12]. For example, a study of Brazilian health system decentralisation, involving anthropological work in three case study areas, investigated the factors shaping the extent of local decision-making actually achieved, with consequences for quality of care improvement possibilities. A range of contextual factors were influential, including political relationships among layers of government, the potential of generating tax revenue at the local level, differences between rural and urban areas in the opportunities for community participation in decision-making, and existing patterns of political patronage; and these also combined with individual management styles and health worker commitment to the local area [13].

In studies with multiple cases, systematic and deliberate cross-case comparison supports, moreover, analytic generalisation (Box 2). The aim in such analysis is not to draw conclusions that can be statistically generalised to a wider study population, or that will hold across time and place. Instead, analytic generalisation entails the development of general conclusions that, although derived from a limited number of particular experiences, provide theoretical insights that can be put forward for consideration, and testing, in other, similar situations. This includes middle range theory, as outlined earlier, and theory that offers ideas about the causal mechanisms likely to underpin interventions that achieve their goals.

**Box 2. An Example of Analytic Generalisation [28]**

A study of the factors underpinning successful family planning programmes involved work in eight country cases. In each country a rich description of the evolution of programme development over time was developed, based on qualitative interviews with policy elites and documentary data analysis.

The countries were paired on the basis of similar socioeconomic development, but in each pair one country had a strong and one a weak, family planning programme. Comparison of experience within and across pairs, suggested that governments' commitment to family planning programmes was influenced by the process of their development and implementation.

More specifically, three factors were identified as likely to underpin successful family planning programmes: coalitions among elite groups with influence over health policy, that support effective programme development; spreading the risk associated with the sensitive issue of family planning among groups and over time; and having a clear and stable organisational structure in charge of implementation, as well as adequate funding. These conclusions were the general insights put forward for consideration and testing in other settings.

### Active Support for Policy Learning

Health research has traditionally seen knowledge generation as essentially a process of adding to the existing stock of facts and predictions, with researchers acting largely as disinterested scientists feeding evidence into the decision-making process [14]. Learning from that knowledge then entails the simple transfer of knowledge from one setting to another [15]. Even current HPSR debates about the importance of getting research into policy and practice and knowledge translation sometimes see this process as quite linear [16].

However, for a relativist, researchers contribute to the process of learning as active participants, using both formal and tacit knowledge in active debate with policy makers [15]. Thus, some social scientists argue that in addressing problems that matter in their own communities, researchers should pay particular attention to the ways in which values and power shape those problems and responses to them [17], assisting policy actors to negotiate mutually acceptable solutions to problems, and ensuring that underrepresented groups are heard [18]. For others, building the possibility of such action into research design is an ethical requirement and key hallmark of good quality research [19].

Social science perspectives, therefore, challenge the HPSR community to think more deeply about how to support policy and system change through their research, including how to address the thorny issue of the boundary between researcher and advocate. For example, what sorts of participatory and action research with citizens, health managers, and health workers can support the reflective enquiry that generates positive change in current practices? And should and can we initiate processes that stimulate public debate about research findings—such as active media engagement, debates on public platforms, or engagement with civil society organisations?

### Ensuring Research Rigour

For some traditions of health research, validity and reliability are the hallmarks of rigorous research, and are ensured through careful study design, appropriate tool development and data collection, and correct approaches to statistical analysis. In contrast, relativist (qualitative) social science research is premised on the understanding that there are multiple realities, reflecting actors' different understandings of common experiences (Figure 1). These understandings are either seen to have significant influence over the issues of focus or to be the focus of inquiry. Researchers from this tradition, moreover, aim not just to identify and report such understandings, but instead, through analysis and engagement, to produce their own interpretations of them, explaining why and how actors behave and think as they do. For relativist research, the “trustworthiness of researchers'” interpretations is the key hallmark of research rigour, implying that the interpretation is widely recognised to have value beyond the particular examples considered. Such trustworthiness is, in essence, negotiated between researchers and research users on the basis of transparent information on study design and the processes of data collection, analysis, and interpretation. Table 1 summarises the critical steps researchers must take to ensure that their analysis is both based on rich insight into the experience examined and has been subject to challenge, and to offer a transparent account of their research process to the user.

**Table 1:** Processes for ensuring rigour in case study and qualitative data collection and analysis [20],[29].

**Principle**	**Example:**A study of the influence of trust in workplace relationships over health worker motivation and performance, involving in-depth inquiry in four case studies [30]
**Prolonged engagement** with the subject of inquiryAlthough ethnographers may spend years in the field, HPSR tends to draw on lengthy and perhaps repeated interviews with respondents, and/or days and weeks of engagement within a case study site	*Case study:*A period of three to four weeks spent in each case study facility*Respondents*Informal engagement & repeated formal interviews
**Use of theory**To guide sample selection, data collection and analysis, and to draw into interpretive analysis	Conceptual framework derived from previous workCase study selection based on assumptions drawn from framework (see below)Theory used in triangulation and negative case analysis (see below)
**Case selection**Purposive selection to allow prior theory and initial assumptions to be tested or to examine “average” or unusual experience	Four primary health care facilities: two pairs of facility types, & in each pair one well and one poorly performing as judged by managers using data on utilization and tacit knowledge (to test assumptions that staff in “well performing” facilities have higher levels of motivation and workplace trust)
**Sampling**Of people, places, times, etc., initially, to include as many as possible of the factors that might influence the behavior of those people central to the topic of focus (subsequently extend in the light of early findings)Gather views from wide range of perspectives and respondents rather than letting one viewpoint dominate	In small case study facilities, interviewed all available staff; in larger facilities, interviewed a purposive sample of staff from each of the staff groups within the facility (considering e.g., age, sex, length of time in facility); interviewed random sample of patients visiting each facility; interviewed all facility supervisors and area manager
**Multiple methods (case studies)**	*For each case study site:*Two sets of formal interviews with all sampled staffResearcher observation & informal discussionInterviews with patientsInterviews with facility supervisors and area managers
**Triangulation**Looking for patterns of convergence and divergence by comparing results across multiple sources of evidence (e.g., across interviewees, and between interview and other data), between researchers, across methodological approaches, with theory	*Within cases:*Initial case reports based on triangulation across all data sets for that case (and across analysts in terms of individual staff members' experience), generating overall judgments about facility-wide experience as well as noting variation in individual health worker experience*Cross-cases:*Initial case reports compared with each other to look for common and different experiences across cases, and also compared with theory to look for convergence or divergence
**Negative case analysis**Looking for evidence that contradicts your explanations and theory, and refining them in response to this evidence	*Within cases:*Triangulation across data identified experiences that contradicted initial assumptions (e.g., about the influence of community interactions over motivation, and about the association between low motivation and poor caring behaviour), and identified unexpected influences (e.g., a general sense of powerlessness among health workers)*Cross-cases:*Cross-site analysis identified facility-level experience that contradicted the initial assumptions underpinning the study (e.g., about the link between high levels of workplace trust, strong health worker motivation, and positive caring behaviour), and identified unexpected conclusions (e.g., about the critical importance of facility-level management over trust and motivation)Report notes weak evidence to support links between levels of workplace trust and client perceptions, but also stronger evidence of links between levels of workplace trust and motivation
**Peer debriefing and support**Review of findings and reports by other researchers	Preliminary case study reports initially reviewed by other members of the research team
**Respondent validation (member checking)**Review of findings and reports by respondents	Preliminary cross-case analysis fed back for review and comment to study respondents; feedback incorporated into final reports
**Clear report of methods of data collection and analysis (audit trail)**Keeping a full record of activities that can be opened to others and presenting a full account of how methods evolved to the research audience	Report provides clear outline of methods and analysis steps as implemented in practice (although on reflection, could be fuller and more reflexive)

At a minimum, improving the quality of HPSR requires paying due attention to the particular approaches to research rigour relevant to the specific paradigm of knowledge underpinning any study. However, because of the complexity of the issues investigated, social science perspectives on rigour offer valuable insights for all empirical HPSR. As HPSR is often more investigation than observation, all stages of research must always be conducted with caution. Rigorous investigation involves the following [19]–[21]:

- an active process of questioning and checking in inquiry—asking how and why things happened and not only what happened, checking answers to questions to identify further issues that need to be followed up to deepen understanding of the experience;
- a constant process of conceptualising and reconceptualising—using ideas and theory to develop an initial understanding of the problem or situation of focus to guide data collection, but using the data collected to challenge those ideas and assumptions and when necessary, to revise your ideas in response to the evidence;
- crafted, interpretative judgements—based on enough evidence, particularly about context, to justify the conclusions drawn, as well as deliberate consideration of contradictory evidence (negative case analysis) and review of initial interpretations by respondents (member checking);
- researcher reflexivity—being explicit about how your own assumptions may influence your interpretation, and testing them in analysis.

Finally, although currently rarely conducted in HPSR, mixed-method research in which qualitative and quantitative analyses are undertaken sequentially, with one stage of work deliberately feeding into the next [22], offer important opportunities for the triangulation across methods and knowledge paradigms that can broaden and deepen investigation of health policy and systems issues [23].

## Conclusions

The current interest in HPSR provides exciting opportunities for the field, but also brings the threat of “disciplinary capture” by the clinical, biomedical, and epidemiological disciplinary perspectives dominant in wider health research. Yet, social science perspectives are vital to HPSR. Health policies and systems are complex social and political phenomena, constructed by human action rather than naturally occurring. Advancing the science of HPSR, thus, demands we take steps to build understanding across disciplinary boundaries, for example, by ensuring that we can speak each other's languages around generalisability and knowledge generation; sharing experience of supporting policy learning; and clarifying expectations of each other's disciplinary culture. Valuing social science perspectives and building interdisciplinary understanding both represents the cutting edge of HPSR and demonstrates that the field is at a scientific cutting edge.

## Abbreviations

HPSR: Health Policy and Systems Research
LMIC: low- and middle-income country
